# *GGT5*: a potential immunotherapy response inhibitor in gastric cancer by modulating GSH metabolism and sustaining memory CD8+ T cell infiltration

**DOI:** 10.1007/s00262-024-03716-3

**Published:** 2024-05-15

**Authors:** Wenjing Zhao, Ziwei Liang, Yongshi Yao, Yang Ge, Guangyu An, Ling Duan, Jiannan Yao

**Affiliations:** 1 Beijing Chaoyang Hospital, Capital Medical University, Beijing, China; 2 Beijing Tiantan Hospital, Capital Medical University, Beijing, China

**Keywords:** *GGT5*, Gastric cancer, GSH, Memory *CD8*+ T cells, Immunotherapy, Immune checkpoints inhibitors

## Abstract

**Purpose:**

The variable responses to immunotherapy observed in gastric cancer (GC) patients can be attributed to the intricate nature of the tumor microenvironment. Glutathione (*GSH*) metabolism significantly influences the initiation and progression of gastric cancer. Consequently, targeting *GSH* metabolism holds promise for improving the effectiveness of Immune checkpoints inhibitors (ICIs).

**Methods:**

We investigated 16 genes related to *GSH* metabolism, sourced from the MSigDB database, using pan-cancer datasets from TCGA. The most representative prognosis-related gene was identified for further analysis. ScRNA-sequencing analysis was used to explore the tumor heterogeneity of GC, and the results were confirmed by  Multiplex immunohistochemistry (mIHC).

**Results:**

Through DEGs, LASSO, univariate and multivariate Cox regression analyses, and survival analysis, we identified *GGT5* as the hub gene in *GSH* metabolism with the potential to promote GC. Combining CIBERSORT, ssGSEA, and scRNA analysis, we constructed the immune architecture of GC. The subpopulations of T cells were isolated, revealing a strong association between *GGT5* and memory *CD8*+ T cells. Furthermore, specimens from 10 GC patients receiving immunotherapy were collected. mIHC was used to assess the expression levels of *GGT5* and memory *CD8*+ T cell markers. Our results established a positive correlation between *GGT5* expression, the enrichment of memory *CD8*+ T cells, and a suboptimal response to immunotherapy.

**Conclusions:**

Our study identifies *GGT5*, a hub gene in *GSH* metabolism, as a potential therapeutic target for inhibiting the response to immunotherapy in GC patients. These findings offer new insights into strategies for optimizing immunotherapy of GC.

**Supplementary Information:**

The online version contains supplementary material available at 10.1007/s00262-024-03716-3.

## Introduction

Gastric cancer (GC) is one of the most prevalent digestive malignancies, ranking fifth in terms of tumor morbidity and fourth in terms of mortality [1]. Its incidence is particularly high in East Asia [2]. Although reports indicate a decline in its incidence rates over the past few years, advanced GC still has an adverse prognosis, with a five-year survival rate of 10% to 30% [3, 4].

In recent years, immunotherapy has shown promise for improving the clinical outcome of patients with GC. However, due to the strong tumor heterogeneity of GC, tumor immune escape events occur frequently, and the efficacy of immunotherapy remains limited and uncertain. According to the results of CheckMate-649 [5], patients with GC who received Nivolumab in combination with chemotherapy had better clinical benefits than those treated with chemotherapy alone, regardless of the *PD-L1* Combined Positive Score (CPS). In the combination treatment group, the two-year overall survival (OS) rate was nearly 40%, progression-free survival (PFS) was significantly increased, and the objective response rate (ORR) was almost 70%. However, the results of KEYNOTE-062 suggested that immunotherapy combined with chemotherapy did not provide any advantage over chemotherapy alone [6]. Therefore, the differences in GC patients' response to immunotherapy need to be addressed. It is essential to investigate the likely mechanisms and determine predictive biomarkers for immunotherapy effectiveness.

The metabolism of tumor cells plays a significant role in regulating the immune response in tumors. Previous studies have already shown that amino acid metabolism is widely involved in immune responses and tumor proliferation in the tumor microenvironment (TME) [7]. Treatment with immune checkpoint inhibitors (ICIs) can enhance the tumor infiltration and anti-tumor function of T cells by reprogramming amino acid metabolism and rebalancing nutrient utilization within the TME [8, 9]. Immunotherapy-induced IFN-γ can inhibit glutathione (*GSH*) synthesis and cause *GSH* depletion, inducing tumor ferroptosis [10]. In order to undertake the large amount of energy required for tumor proliferation, invasion, and metastasis, tumor cells significantly enhance the uptake and catabolism of glutamine, competing with T cells [11, 12]. Tumor cells can regulate reactive oxygen species (*ROS*) metabolism through *GSH* and *NADPH* produced by glutamine metabolism to maintain redox homeostasis [13]. Blocking *GSH* metabolism can not only inhibit tumor proliferation, but also restore anti-tumor immunity. Inhibition of *GSH* metabolism leads to a decrease in myeloid-derived suppressor cells (MDSCs) and facilitates their conversion into M1 macrophages, which promote the process of cross-presentation for tumor antigens and activate *CD8*+ T cells, thereby performing anti-tumor functions [14]. Therefore, targeting *GSH* metabolism has the potential to improve immunotherapy response and survival outcomes in GC patients as a novel strategy. However, the mechanism of *GSH* metabolism in regulating TME in GC patients requires further exploration.

Considering the issues mentioned above, we investigated genes related to *GSH* metabolism in the Molecular Signatures Database (MSigDB) and used them to construct a prognostic gene signature based on data from The Cancer Genome Atlas (TCGA) public database. To understand the potential mechanism of immune evasion in GC, we conducted CIBERSORT analysis and tumor immune dysfunction and exclusion (TIDE) analysis. Finally, we conducted a systematic interrogation of different immune cell types related to hub genes through single-cell RNA (scRNA) sequencing analysis, aiming to elucidate the critical role of immune cells related to *GSH* metabolism in regulating tumor progression in GC. These findings should provide potential strategies to enhance the response to immunotherapy in GC.

## Materials and methods

### Data source

The bulk RNA-seq data (HTSeq-count format) and clinical information for TCGA, which includes 11,069 samples from 33 types of cancer, were downloaded from the Xena (https://xenabrowser.net/). The external validation cohort of GC was acquired from GSE15459 [15], included 200 primary gastric tumors based on GPL570 platform (Affymetrix Human Genome U133 Plus 2.0 Array). The single-cell dataset (GSE167297 [16]) was obtained from the GEO database, which includes 15,729 cells from 10 human superficial and deep layers of diffuse-type gastric cancer using 10X Genomics. Gene sets related to *GSH* metabolism were retrieved from the MSigDB database [17] (https://www.gsea-msigdb.org/gsea/msigdb). Three pathways, namely Kyoto Encyclopedia of Genes and Genomes (KEGG), WikiPathways (WP), and Gene Ontology Biological Process (GOBP), were found to be highly correlated. To further explore the gene signatures of *GSH* metabolism, we performed an intersection of the three gene sets. All statistical analysis of data was performed using R software (version 4.1.3).

### Differential expression analysis and functional enrichment analysis

We compared the differential expression level of gene signatures involving GSH metabolism across cancer types based on the count format data from TCGA database, using the R package "limma." Marker genes were then identified by setting the threshold at a false discovery rate (FDR) < 0.05. To explore highly correlated pathways, we performed GO and KEGG functional enrichment analyses using the R package "clusterProfiler" and filtered the GO and KEGG terms with a cutoff of *p* value < 0.05 and FDR < 0.05. Finally, we created a circle plot using the R package "GOplot."

### Construction of a prognostic model

The "glmnet" R package was used to conduct Least Absolute Shrinkage and Selection Operator (LASSO) Cox regression analysis to select genes significantly associated with GC prognosis. Univariate and multivariate Cox regression analyses were also performed to screen the most independent prognostic factors for GC patients. Ultimately, *GGT5* was identified as the hub gene for GSH metabolism in GC, as it was differentially expressed in both tumor and normal tissues and emerged as an independent prognostic factor. The optimal cutoff value for *GGT5* was determined using the "surv_cutpoint" function in the "survminer" R package. Patients with GC were then divided into high and low group based on this cutoff value, and Kaplan-Meier (KM) survival curves and the log-rank test were constructed using the "survival" and "survminer" R packages. The HR value was calculated using the “survival” R package. RNA-seq data of 200 gastric cancer patients and their survival data affiliated from GSE15459 were analyzed for external validation. Patients were divided into high and low groups according to the optimal cutoff value of *GGT5*; then, the KM survival curve was plotted.

Clinical information on gastric cancer was collected from TCGA, including tumor stage, histological grade, T stage, N stage, and M stage. Based on these clinical characteristics, patients were clustered into different risk groups. The expression level of *GGT5* was measured in each group and plotted in a violin plot using the "ggplot2" package.

DCA (Decision Curve Analysis) is a method for assessing the net benefit of a forecasting model. We conducted the DCA curve to predict the clinic benefit of *GGT5* signature compared with another four published models using the “ggDCA” R package.

### Estimation of immune cell infiltration

CIBERSORT analysis was used to identify differential infiltration of immune cells between groups with high/low *GGT5* expression. Spearman correlation analysis was then employed to explore immune cell infiltration. The "ggpubr" package was utilized to plot immune cell abundance using a violin plot. Immune cells with statistically significant differences were selected for visualization in scatter plots using the "ggplot2" package. MCP-counter allows quantification of the absolute abundance of 8 immune cell populations and 2 stromal cell populations in a mixed tissue from transcriptome data. EPIC (Estimating the Proportion of Immune and Cancer cells) can accurately detect major cell types in tumors directly based on gene expression levels in tumors. xCell combines the ssGSEA analysis with deconvolution methods to understand the heterogeneity of cells that make up the tumor microenvironment in tumor samples. ESTIMATE infers the ratio of stroma and immune cells in a tumor sample from gene expression characteristics. In our study, we conducted MCP-counter, EPIC, xCell, ESTIMATE analysis to validate the result of CIBERSORT based on the “IOBR” R package.

Additionally, we obtained markers of 28 different immune cell types from the TISIDB [18] database (http://cis.hku.hk/TISIDB), an online portal for studying tumor and immune system interactions. Single-sample gene set enrichment analysis (ssGSEA) was used to investigate the relationship between hub genes and infiltration of the 28 immune cell types using the "GSVA" package.

### Tumor immune dysfunction and exclusion (TIDE) and the relationship with immune-related genes

To investigate immune evasion mechanisms in GC, we examined the dysfunction and exclusion of T cells. We employed TIDE analysis to predict the curative effect of immunotherapy. Patients with higher TIDE scores are more likely to experience immune escape and have a poorer response to ICIs. For a deeper understanding of the close relationship between *GGT5* and immune infiltration, we visualized the correlation between *GGT5* and immune-related genes, such as immune activation genes, immunosuppressive genes, chemokines, and chemokine receptors, using a bubble plot generated by the "corrplot" package.

### ScRNA data processing and dimensionality reduction

The GC scRNA-seq data used in this study were obtained from GSE167297, which was published in the GEO database. The dataset consisted of 10 samples, and all raw data were processed using the "Seurat" package. Quality control was performed according to the following criteria: (1) the cell count of each sample was at least 500, and (2) the proportion of mitochondrial genes was less than 5%. Next, the "NormalizeData" function was used to normalize the s4 data, and the "FindVariableFeatures" function was applied to recognize the top 2000 highly variable genes (HVGs). The selected HVGs were further identified, centered, and scaled using the "ScaleData" function.

### Cell clustering, annotation, and marker genes identification

According to principal component analysis (PCA), scores were assigned to the Seurat data. Each PC term included half of its features, which combined information related to the features. The "RunPCA" function was used for dimension reduction of highly variable genes (HVGs). Spectral clustering was then employed to identify different cell clusters. The "FindNeighbors" and "FindClusters" functions were subsequently utilized for further clustering, with the resolution parameter set to 0.4. Nonlinear dimensionality reduction was visualized by T-Distributed Random Neighbor Embedding (t-SNE). To cluster different cell types, the "SingleR" package was used to reference a database. The "FindAllMakers" function was then employed to screen for characterized genes with a logfc.threshold of 0.5. The expression level of *GGT5* in each different cell type was measured and portrayed as a violin plot. The composition ratio and cell number of each cell type were compared and displayed in a bar chart using the "ggplot2" package. T cells were extracted for further analysis. The NormalizeData, RunPCA, FindNeighbors, and FindClusters functions were reloaded to cluster different T cell subtypes. The tSNE clustering analysis was reperformed to display the expression distribution of T cell subsets. Marker genes for each subgroup of T cells were screened in published articles and the CellMarker database, and the identified clusters were reannotated. The expression value of *GGT5* in all T cell subsets and the correlation scores between *GGT5* and different *CD8* T cells were calculated.

### Pseudotime trajectory analysis

To classify the exhaustion of T cells during the process of tumor-educating, the “Monocle 2” package was utilized for trajectory of each single cell. The raw UMI count matrices of 10 gastric cancer patients were imported using Seurat and converted as a cds file with Monocle. Following the standard quality control process, we filtered some low-quality cells, setting the filter parameters as follows: mean expression ≥ 0.1, num_cells_expressed ≥ 10, the q-value of different genes was less than 0.05. Then, we portrayed the trajectory of T cells, CD8+ T cells, and CD4+ T cells into tSNE plots.

### Multiplex immunohistochemistry (mIHC) analysis

To investigate the connection between *GGT5* expression and memory *CD8*+ T cells, and the response to immunotherapy, a total of 10 specimens of GC patients undergoing immunotherapy at Beijing Chaoyang Hospital were obtained for this study. Each patient's response to immunotherapy was evaluated based on RECIST criteria, which categorized the patients into either the partial response (PR) group or the non-PR group. mIHC analysis was performed with the Opal™ 4-color Multiplex reagents kit (PerkinElmer, USA). Antibodies for *GGT5* (ab283267), *CD45RO* (ab23), *CCR7* (ab253187) were purchased from Abcam Inc. Antibody for *CD8* (66868-1-Ig) was purchased from Proteintech. According to the manufacturer’s instructions, nuclear staining was conducted with *DAPI* (PerkinElmer, USA), *GGT5* (ab283267, 1:2000) was stained with opal 620, *CD8* (66868-1-Ig, 1:50,000) was stained with opal 570, *CD45RO* (ab23, 1:5000) was stained with opal 520, *CCR7* (ab253187, 1:300) was stained with opal 690. Memory *CD8*+ T cells were defined as *CD8*, *CD45RO*, and *CCR7*-positive cells. The multiplex fluorophore-stained slides were scanned by Beijing Bodu Hengyi Technology company. Each channel was individually captured and analyzed using NDP View 2 software.

## Result

### Construction of *GSH* metabolic signature

**Fig. 1:**
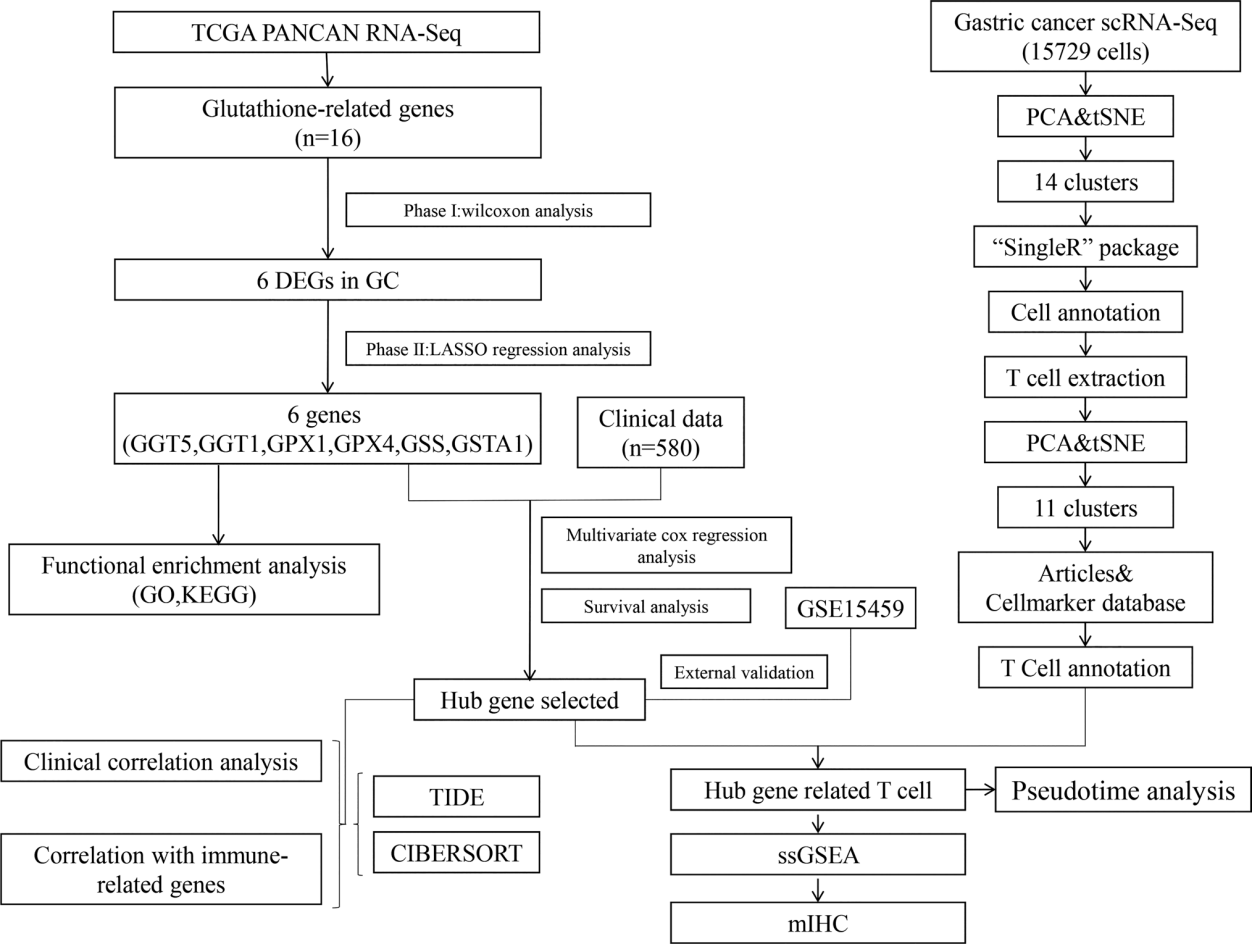
Analysis workflow of this study

**Fig. 2:**
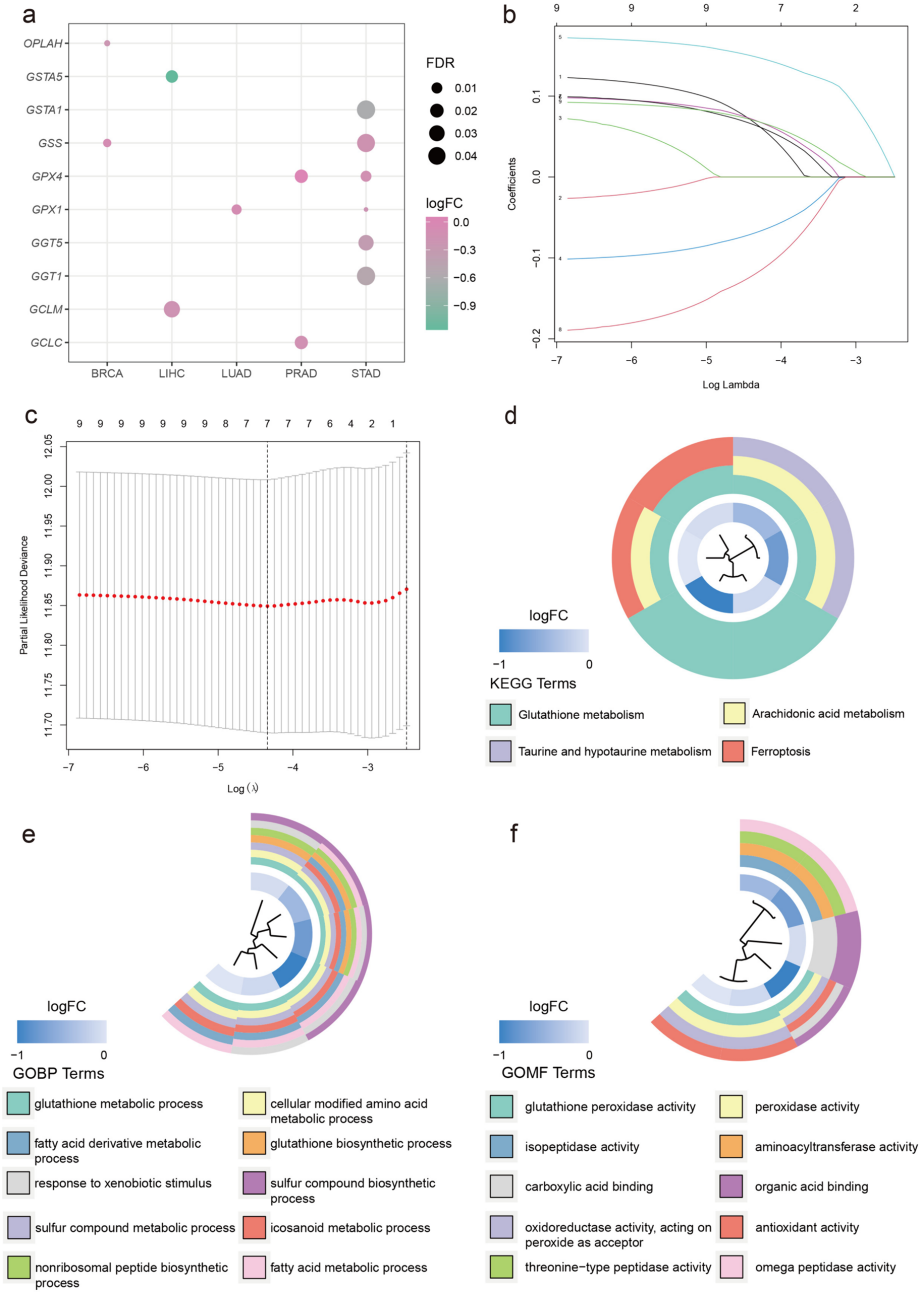
The expression mode diagram of glutathione metabolism-related genes of pan-cancer from TCGA and enrichment of GO and KEGG **a** The bubble plot displays the differentially expression diagram of genes associated with glutathione metabolism between tumor and normal tissues across pan-cancer types. Only the data of FDR < 0.05 were retained and it shows that there are 6 genes differentially expressed in GC. The dot size indicates the FDR and the color represents the fold-change, **b**, **c** LASSO regression analysis to screen the prognostic-related genes, **d** the circle plot for KEGG analysis, terms with *p* < 0.05 were present. The top 10 terms of BP (**e**) and MF (**f**) for GO analysis, of which the *p* value is less than 0.05, were plotted

The workflow chart of this study is presented in Fig. 1. Initially, the MSigDB database was searched to systematically identify signature genes of *GSH* metabolism. By intersecting the 3 *GSH* metabolism gene sets from KEGG, WK, and GOBP databases, a total of 16 genes were selected. Next, these genes were analyzed in 33 types of pan-cancer datasets from TCGA. After excluding cancer types without matched normal samples, 17 cancer types were retained for further analysis, and differential expression gene (DEG) analysis of GSH metabolic genes was performed between the 17 cancer types and normal tissues (Fig. S1a). Of these, 6 *GSH* metabolism genes were found out to be differentially expressed in GC (*p* < 0.05) (Fig. 2a). These genes included *GGT1*, *GGT5*, *GPX1*, *GPX4*, *GSS*, and *GSTA1* (Table 1). Furthermore, LASSO regression analysis was conducted to screen potential prognostic-related genes (Fig. 2b, c), and all six of these genes were found to be prognostic factors and were considered as *GSH* metabolic signature.

**Table 1:** Information of 6 glutathione metabolic key genes

Gene	Full name of gene	Coefficient	Metabolism-related KEGG pathways
*GGT5*	Gamma-glutamyltransferase 5	0.15532	Glutathione metabolism Taurine and hypotaurine metabolism Arachidonic acid metabolism Metabolic pathways
*GPX4*	Glutathione peroxidase 4	0.108807	Glutathione metabolism Arachidonic acid metabolism Metabolic pathways Ferroptosis
*GPX1*	Glutathione peroxidase 1	0.093588	Glutathione metabolism Metabolic pathways Thyroid hormone synthesis Amyotrophic lateral sclerosis Huntington disease
*GSTA1*	Glutathione S-transferase alpha 1	0.089869	Glutathione metabolism Metabolism of xenobiotics by cytochrome P450 Drug metabolism—cytochrome P450
*GGT1*	Gamma-glutamyltransferase 1	-0.10619	Glutathione metabolism Taurine and hypotaurine metabolism Cyanoamino acid metabolism Arachidonic acid metabolism
*GSS*	Glutathione synthetase	-0.17165	Glutathione metabolism Cysteine and methionine metabolism Biosynthesis of cofactors Ferroptosis

### GO and KEGG enrichment analysis of key genes

For a deeper understanding of the correlation of the signature and GC, the ‘clusterProfiler’ package was utilized to perform GO and KEGG analysis. The results are presented in Fig. 2d–f. For BP, MF, and KEGG, six of the selected key genes were strongly correlated with *GSH* metabolism, which validates the screening process. Additionally, KEGG analysis revealed enrichment in arachidonic acid metabolism, taurine and hypotaurine metabolism, and ferroptosis. GO BP terms showed that the top two terms were cellular modified amino metabolic process and sulfur compound metabolic process. In GO MF terms, the top two terms were peroxidase activity and oxidoreductase activity, acting on peroxide as acceptor.

### Identification of the key gene of *GSH* metabolism in GC

**Fig. 3:**
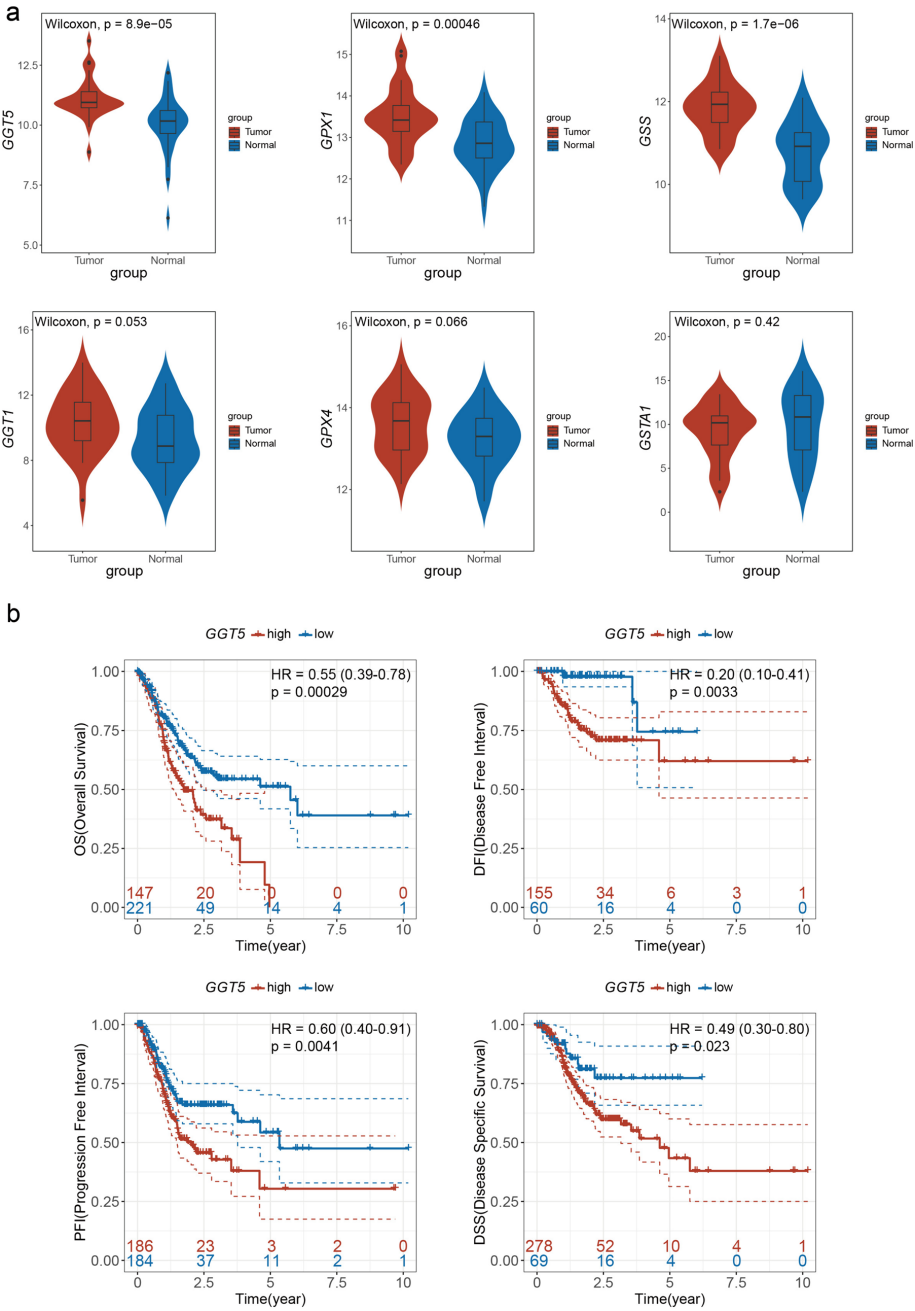
Expression pattern and survival analysis of the key genes for GSH metabolism **a** The differential expression level of 6 glutathione metabolic signature in GC and adjacent normal tissues shows that expression values of *GGT5*, *GPX1*, and *GSS* are extremely higher in gastric cancer (*p* < 0.05), while the expression values of *GGT1*, *GPX4*, and *GSTA1* have no significant meaning between GC and adjacent normal tissues, **b** the GC samples were classified into high and low groups of *GGT5* expression according to the maxstat method. The KM curves including overall survival analysis (*p* = 0.00029), progression-free interval analysis (0.0041), disease-free interval analysis (*p* = 0.0033), and disease-specific survival analysis (*p* = 0.023) were plotted, and these suggest that *GGT5* is a risk gene associated with a poor outcome of gastric cancer

To explore the latent mechanism of the key genes in GC, the differential expression levels between GC and paired adjacent normal tissues were measured using ‘limma’ package. The results were presented as violin plots using the ‘ggplot’ package (Fig. 3a). The expression values of *GGT5*, *GPX1*, and *GSS* were found to be significantly higher in GC (*p* < 0.05), whereas the expression values of *GGT1*, *GPX4*, and *GSTA1* showed no statistical significance between GC and normal tissues. Furthermore, univariate Cox regression analysis was conducted to determine the prognostic effect (Table 2). This study included age, gender, histologic grade, tumor stage, T stage, N stage, M stage, six key genes, and risk score calculated by LASSO analysis, radiation therapy, reflux history, antireflux treatment, Barrett's esophagus, family history, new tumor event, lymph node count, lymph nodes positive, and signet ring cell carcinoma to identify the risk factors of GC. According to the results, *GGT5* was identified as the hub gene for GSH metabolism in GC. Not only was it differentially expressed in tumor tissue, but it also emerged as an independent prognostic gene.

**Table 2:** Univariate and multivariate Cox regression analysis of 6 glutathione metabolic key genes and clinic characteristics in gastric cancer

Variables		Univariate analysis		Multivariate analysis	
		HR (95% CI)	*p* value	HR (95% CI)	*p* value
Age	> = 65/ < 65	1.6 (1.1–2.2)	0.01	1.82 (1.23–2.69)	0.003
Gender	Female/male	1.3 (0.93–1.9)	0.12		
histologic.grade	G3/G1 + G2	1.3 (0.93–1.9)	0.121	1.27 (0.85–1.90)	0.24
tumor.stage	III + IV/I + II	1.8 (1.3–2.6)	0.001	1.25 (0.64–2.45)	0.506
M.stage	M1/M0	2.0 (1.15–3.6)	0.015	1.59 (0.84–3.01)	0.155
N.stage	N2 + N3/N1	1.6 (1.15–2.2)	0.005	1.41 (0.86–2.29)	0.171
T.stage	T3 + T4/T1 + T2	1.6 (1.1–2.5)	0.018	1.41 (0.83–2.42)	0.206
*GGT1*		0.92 (0.8–1.1)	0.275	0.86 (0.72–1.03)	0.109
*GGT5*		1.2 (1–1.5)	0.013	1.21 (0.98–1.48)	0.07
*GPX1*		1.2 (0.94–1.5)	0.165	1.03 (0.74–1.42)	0.878
*GPX4*		1.2 (0.91–1.5)	0.23	1.01 (0.72–1.42)	0.939
*GSS*		0.82 (0.64–1.1)	0.129	0.84 (0.59–1.20)	0.339
*GSTA1*		1.1 (0.99–1.1)	0.112	1.09 (1.00–1.20)	0.055
riskScore		2.8 (1.6–4.9)	< 0.001		
radiation.therapy	Yes/No	0.33 (0.16–0.69)	0.003	0.43 (0.20–0.92)	0.029
reflux.history	Yes/No	0.58 (0.29–1.2)	0.128		
antireflux.treatment	Yes/No	0.76 (0.43–1.4)	0.361		
barretts.esophagus	Yes/No	1.1 (0.44–2.7)	0.842		
family.history	Yes/No	1.0 (0.49–2.1)	0.978		
new.tumor.event	Yes/No	3.7 (2.4–5.6)	< 0.001	3.51 (2.18–5.64)	< 0.001
lymph.node.count	≥ 16/< 16	0.81 (0.51–1.1)	0.241		
lymphnodes.positive	Yes/No	2.0 (1.3–3.1)	0.002	0.98 (0.52–1.87)	0.961
Signet.ring.cell.carcinoma	Yes/No	2 (0.99–4.1)	0.052		

### Clinical characteristics and outcome related to *GGT5* in gastric cancer

To estimate the prognostic value of *GGT5*, KM curves were portrayed to display the survival rate of GC patients. The results revealed that patients in group of high *GGT5* expression had poorer overall survival (OS) than those with low expression (*p* = 0.00029, HR (95% CI) 0.55 (0.39–0.78)). Similarly, the progression-free interval (PFI) (*p* = 0.0041, HR (95% CI) 0.60 (0.40–0.91)), disease-free interval (DFI) (*p* = 0.0033, HR (95% CI) 0.20(0.10–0.41)), and disease-specific survival (DSS) (*p* = 0.023, HR (95% CI) 0.49 (0.30–0.80)) consistently showed a survival trend (Fig. 3b), indicating that higher expression of *GGT5* was closely related to adverse clinical outcomes in GC patients. To validate the result in the TCGA cohort, we collected 200 GC patients from GSE15459; it shows that high *GGT5* expression group had a significantly worse OS than the low *GGT5* expression group (Fig. S3a, *p* = 0.00035, HR (95% CI) 0.49(0.32–0.76)).

Besides, DCA curves were used to compare our *GGT5* model with another four published models [19–22]. We found that *GGT5* has better clinical application value than other GC signatures (Fig. S3b). Furthermore, the correlation between *GGT5* and clinical–pathologic characteristics, including histologic grade, tumor stage, T stage, N stage, and M stage, was measured. According to T stage and histologic grade, *GGT5* was expressed at higher levels in T3 + T4 stages than in T1 + T2 stages (*p* = 0.006) and in G3 grade than in G1 + G2 grades (*p* = 0.00014), indicating that the value of *GGT5* increased with higher clinical stage (Fig. 5a). However, the results between *GGT5* and tumor stage, N stage, and M stage showed no statistical significance (Fig. S2b). In conclusion, these findings show that patients with higher expression of *GGT5* have poorer clinical outcomes.

### Correlation Between *GGT5* and Immune Infiltration

**Fig. 4:**
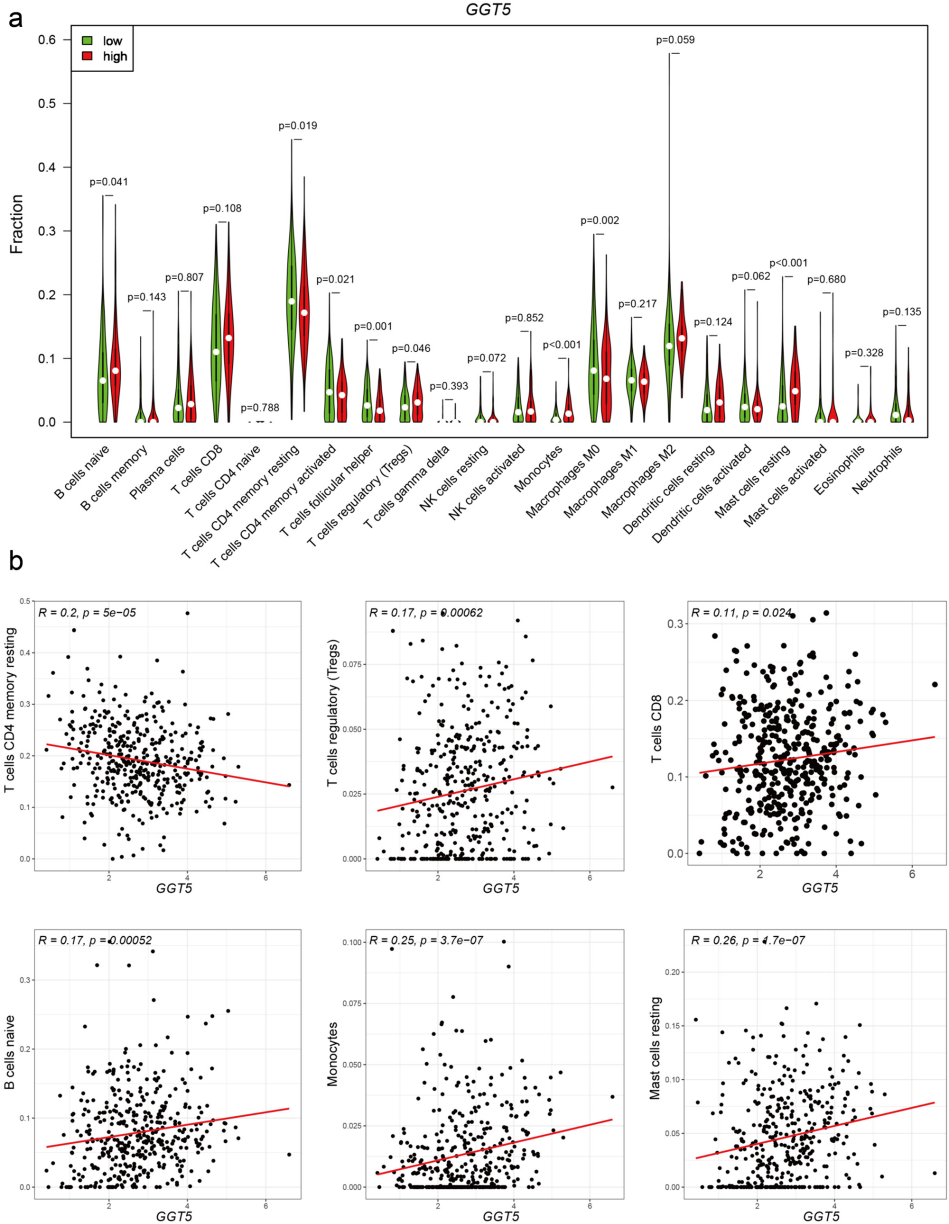
The relationship between *GGT5* and immune cells of GC **a** CIBERSORT analysis reveals the relationship of *GGT5* with 22 kinds of immune infiltrating cells, it indicates that there are 8 kinds of immune cells differentially expressed in high (red) and low (green) expression group of *GGT5* (*p* < 0.05), **b** the correlation analysis displays a positive connection between *GGT5* with T cells regulatory (Treg), T cells *CD8*, B cells naïve, monocytes, and mast cells resting, while it has a negative connection with T cells *CD4* memory resting

The proportion of tumor-infiltrating immune subsets was employed according to the CIBERSORT algorithm, by deconvoluting the expression matrix of 22 immune cells through linear support vector regression [23, 24]. In our study, the GC samples were classified into high/low groups based on the optimal cutoff value of *GGT5* and subsequently analyzed for tumor heterogeneity between the two groups. As depicted in Fig. 4a, naïve B cells (*p* = 0.041), regulatory T cells (Tregs) (*p* = 0.046), monocytes (*p* < 0.001), and resting Mast cells (*p* < 0.001) were observed to have higher expression levels in the high *GGT5* group, whereas the low expression group exhibited high expression levels of resting *CD4* memory T cells (*p* = 0.019), activated *CD4* memory T cells (*p* = 0.021), follicular helper T cells (*p* = 0.001), and M0 macrophages (*p* = 0.002). EPIC, MCP-counter, xCell, and ESTIMATE were conducted to validate the results above. All of these methods to explore the immune infiltration mode of tumor microenvironment show consistent trend; the CD8+ T cells were observed to have significantly higher expression levels in the GGT5 high expression group (Fig. S3c-d).

For further validation, we calculated the correlation value of *GGT5* and various immune cells, as illustrated in Fig. 4b. Our analysis revealed that *GGT5* exhibited a positive correlation with regulatory T cells (Tregs) (R = 0.17, *p* = 0.00062), *CD8* T cells (R = 0.11, *p* = 0.024), naïve B cells (R = 0.17, *p* = 0.00052), monocytes (R = 0.25, *p* = 3.7e−07), and resting Mast cells (R = 0.26, *p* = 1.7e−07). Conversely, *GGT5* showed a negative association with resting *CD4* memory T cells (R = -0.2, *p* = 5e−05). However, the correlation between *GGT5* and immune cells like activated *CD4* memory T cells, follicular helper T cells, and M0 macrophages was not statistically significant (Fig. S2a). These findings suggest that *GGT5* may influence different types of immune cells, particularly T cells, to modulate the TME of GC.

### Relationship between *GGT5* and immune-related genes and immune therapy

**Fig. 5:**
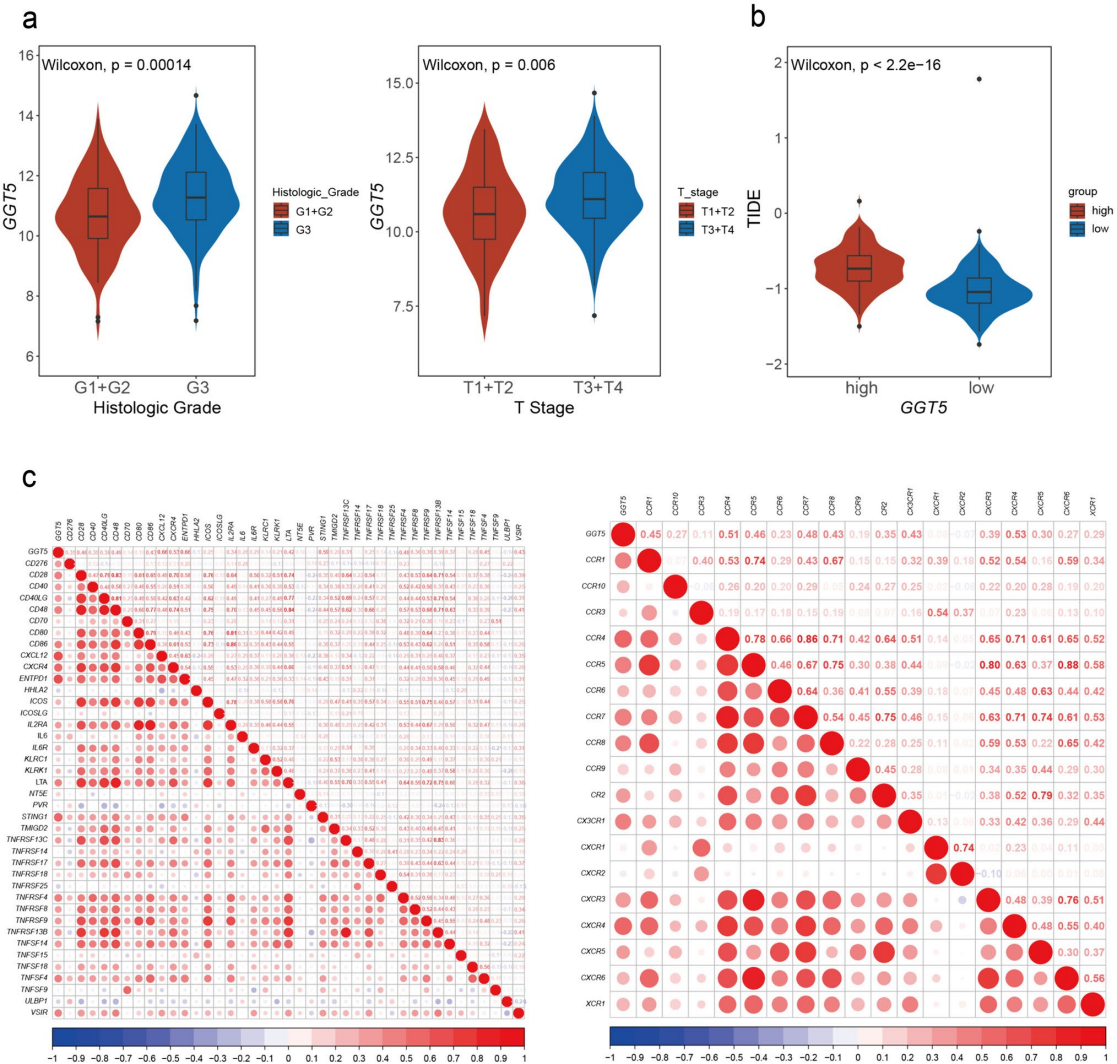
The relationship of *GGT5* with clinicopathological characters and immune features **a** the relationship between *GGT5* with clinicopathological characters of gastric cancer, including histologic grades (*p* = 2.1e−06) and T stage (*p* = 0.018), indicates that high expression of *GGT5* is highly related to the development of gastric cancer, **b** the prediction efficiency of response to ICIs based on TIDE scores shows that higher expression of *GGT5* is related to a higher TIDE score, also a poorer response to ICIs therapy, **c** the bubble plots represent the relationship of *GGT5* with immune-related genes, including immune-active genes and chemokine receptors. It displays that *GGT5* is positively associated with these immune-related genes. The color and size of the dots represent the correlation coefficient

The TIDE scores were utilized to predict the efficiency of immunotherapy on *GGT5*. A higher TIDE score indicates a greater tendency of immune escape and a poorer response to immune checkpoint inhibitors (ICIs). As shown in Fig. 5b, patients with higher expression of *GGT5* had a higher TIDE score (*p* < 2.2e−16), suggesting that *GGT5* overexpression may lead to an adverse response to immunotherapy. To further investigate the role *GGT5* in the TME of GC, correlation analysis between *GGT5* and immune genes was estimated (Fig. 5c). The results show a positive correlation with immune active genes, immune suppressive genes, chemokines, and chemokine receptors, particularly immune active genes and chemokine receptors. These findings suggest the regulatory role for *GGT5* in the TME of GC is possibly related to these immune-related genes.

### Identification of *GGT5*-related immune cell infiltration in gastric cancer

**Fig. 6:**
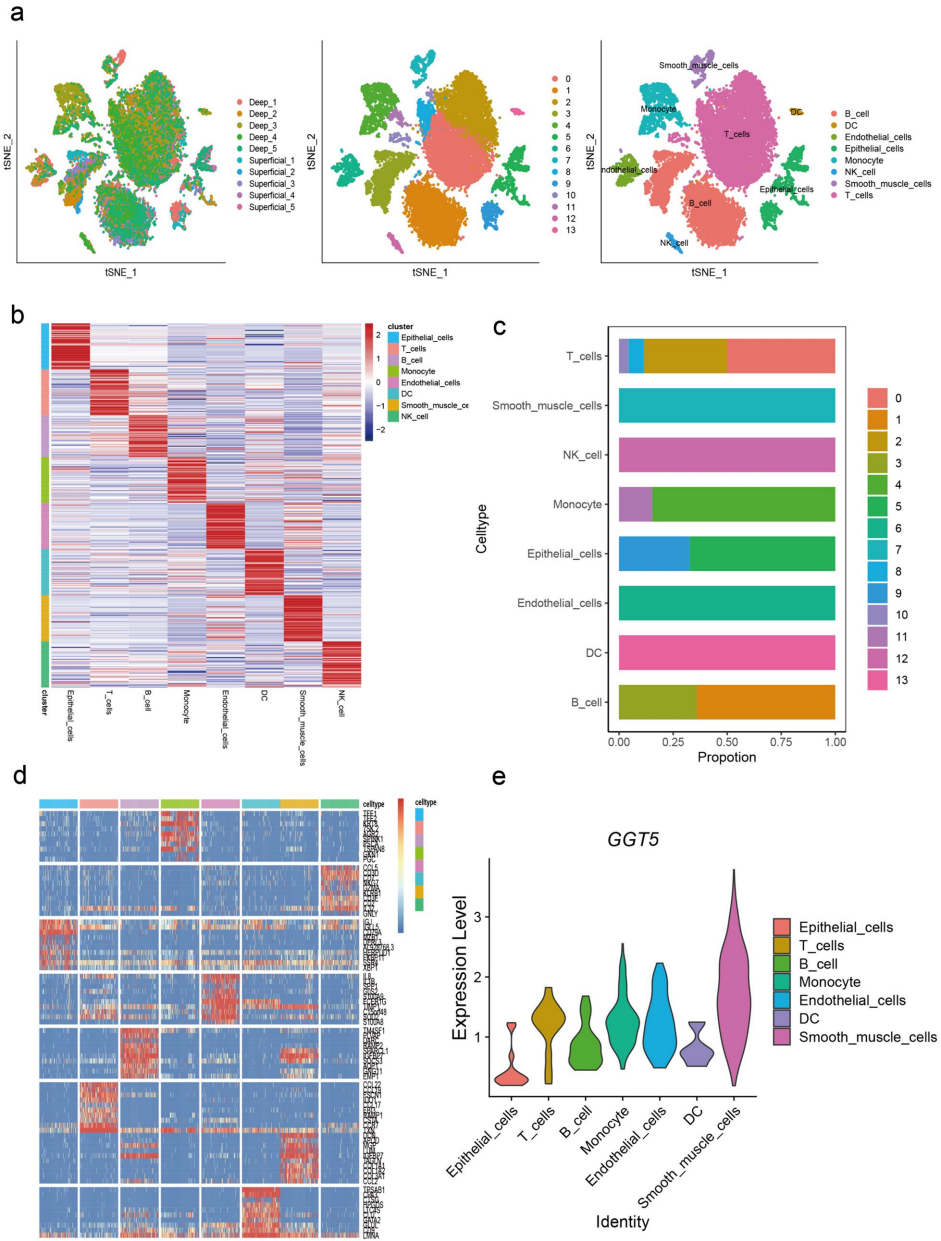
Overview of single-cell analysis from gastric cancer **a** a demonstration of t-SNE of gastric cancer samples, 14 cell clusters, and different cell types identified by marker genes, **b** the expression pattern of each cell type, **c** the cell ratio in each cluster indicates that T cell has the most multiple subsets, **d** heatmap shows the top 10 marker genes of clusters, representing the characteristic features of each cell type, **e** the expression level of *GGT5* in each cell type shows that *GGT5* has a high expression value in T cells

For this study, we analyzed 10 gastric cancer samples from GSE167297. After adjusting for batch differences, we integrated the samples and identified the top 2000 highly variable genes (HVGs) based on their mean and dispersion. We then performed principal component analysis (PCA) downsizing and retained the top 20 dimensions of cells. Next, we used t-SNE clustering to visualize the clustering of 15,729 cells from gastric cancer tissues, which were assigned to 14 clusters (Fig. 6a). Using the "SingleR" package and marker genes, we identified 8 cell types, including T cells, B cells, dendritic cells (DC), endothelial cells, epithelial cells, monocytes, NK cells, and smooth muscle cells (Fig. 6b). Notably, T cells had multiple subgroups and a high number of cells, indicating their potential importance in gastric cancer (Fig. 6c).

To validate our findings, we screened the top 10 HVGs of each cluster (Fig. 6d), which were confirmed as marker genes for each cell type. We then portrayed the expression level of *GGT5* in each infiltrating cell type using a violin plot (Fig. 6e), which showed that *GGT5* expressed particularly higher in T cells. Therefore, we selected T cells for further investigation and sub-division in this study.

**Fig. 7:**
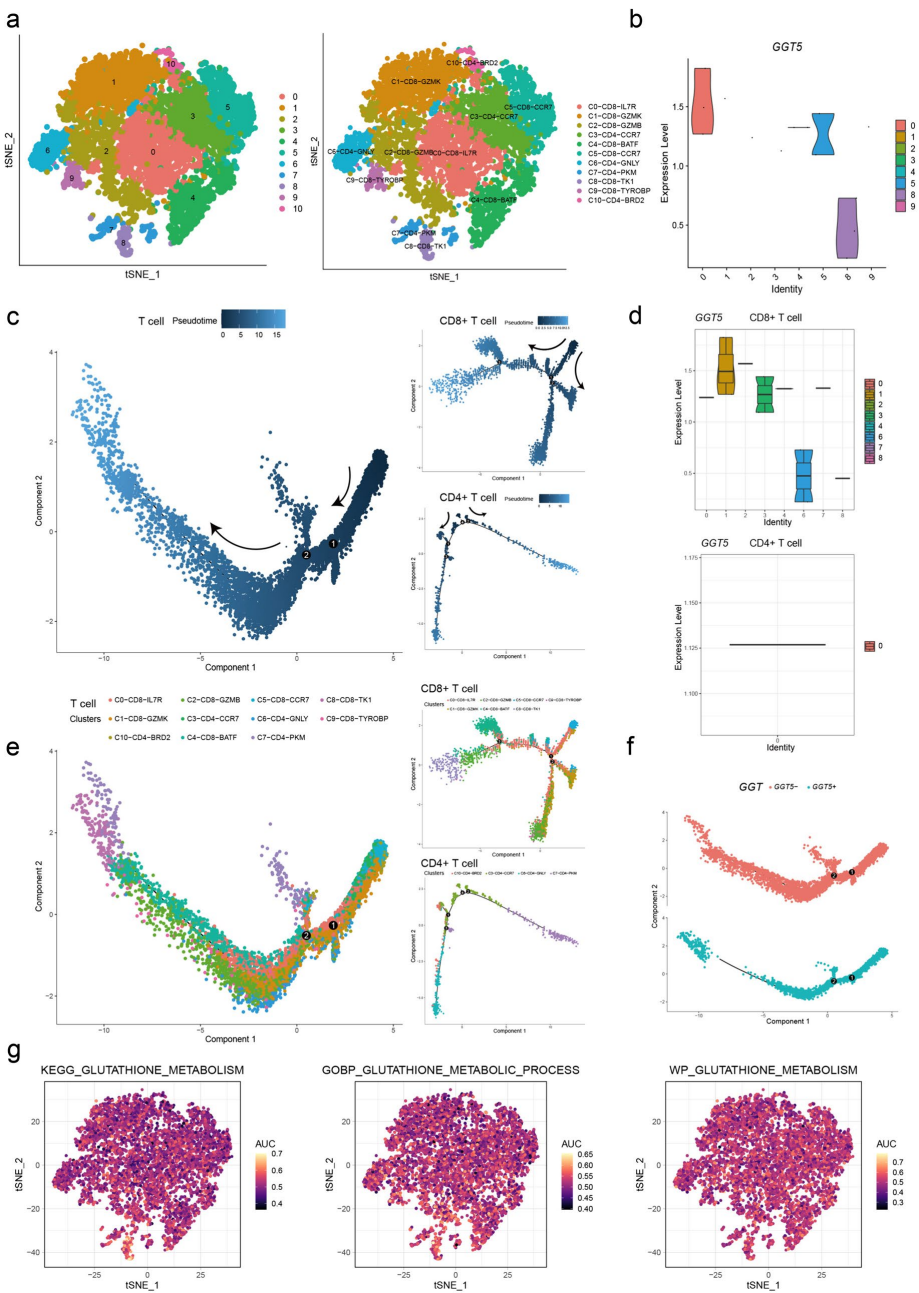
Overview of single cells from T cells **a** the t-SNE plot shows 11 clusters and different subsets of T cells, **b** the expression value of *GGT5* in each subgroup of T cells indicates that *GGT5* is highly expressed in *C0-CD8-IL7R*, *C5-CD8-CCR7*, and *C8-CD8-TK1*. **c**, **d** different trajectories of T cells, *CD8*+ T cells, and *CD4*+ T cells, with the shade and color for the pseudotime and clusters calculated by monocle, **e** the expression level of *GGT5* in *CD8*+ T cells and *CD4*+ T cells, **f** pseudotime analysis of *GGT5* ± T cells, **g** the degree of activity of 3 GSH metabolism gene sets from the MSigDB database based on the AUC value shows that glutathione metabolism is actively expressed in T cells

We performed PCA downsizing again for selected T cells, retaining only the top 10 dimensions. This led to the identification of 11 clusters (Fig. 7a), which were reannotated using marker genes highly expressed in each cluster and lowly expressed in others, based on the CellMarker database and relevant articles. Next, we measured the activity of GSH metabolism using the KEGG, GO, and WikiPathways databases (Fig. 7g) and found that it was active in T cells of gastric cancer. To increase our understanding of *GGT5*’s role in T cells, we measured the expression value of *GGT5* in each subpopulation available (Fig. 7b) and found that it was most highly expressed in *C0-CD8-IL7R*, *C5-CD8-CCR7*, and *C8-CD8-TK1* clusters, suggesting a close association between *GGT5* and *CD8* T cells. To evaluate the differentiation states of different T cells, the continuous trajectory of T cell exhaustion was portrayed by Monocle 2 and visualized as a tSNE plot (Fig. 7c). Additionally, we isolated the *CD8* and *CD4* T cell clusters to elucidate the association between *GGT5* and *CD8*+ T cells. The results indicate that cluster 0 and 5 were predominantly observed in the early and middle stages of the differentiation trajectory for both the entire T cell exhaustion system and *CD8*+ T cells (Fig. 7d). The expression level of *GGT5* in *CD8*+  T cells was significantly higher than in *CD4*+ T cells (Fig. 7e), with concentration primarily in the early and middle phases of the T cell trajectory (Fig. 7f). This suggests that elevated *GGT5* expression might contribute to T cell exhaustion.

**Fig. 8:**
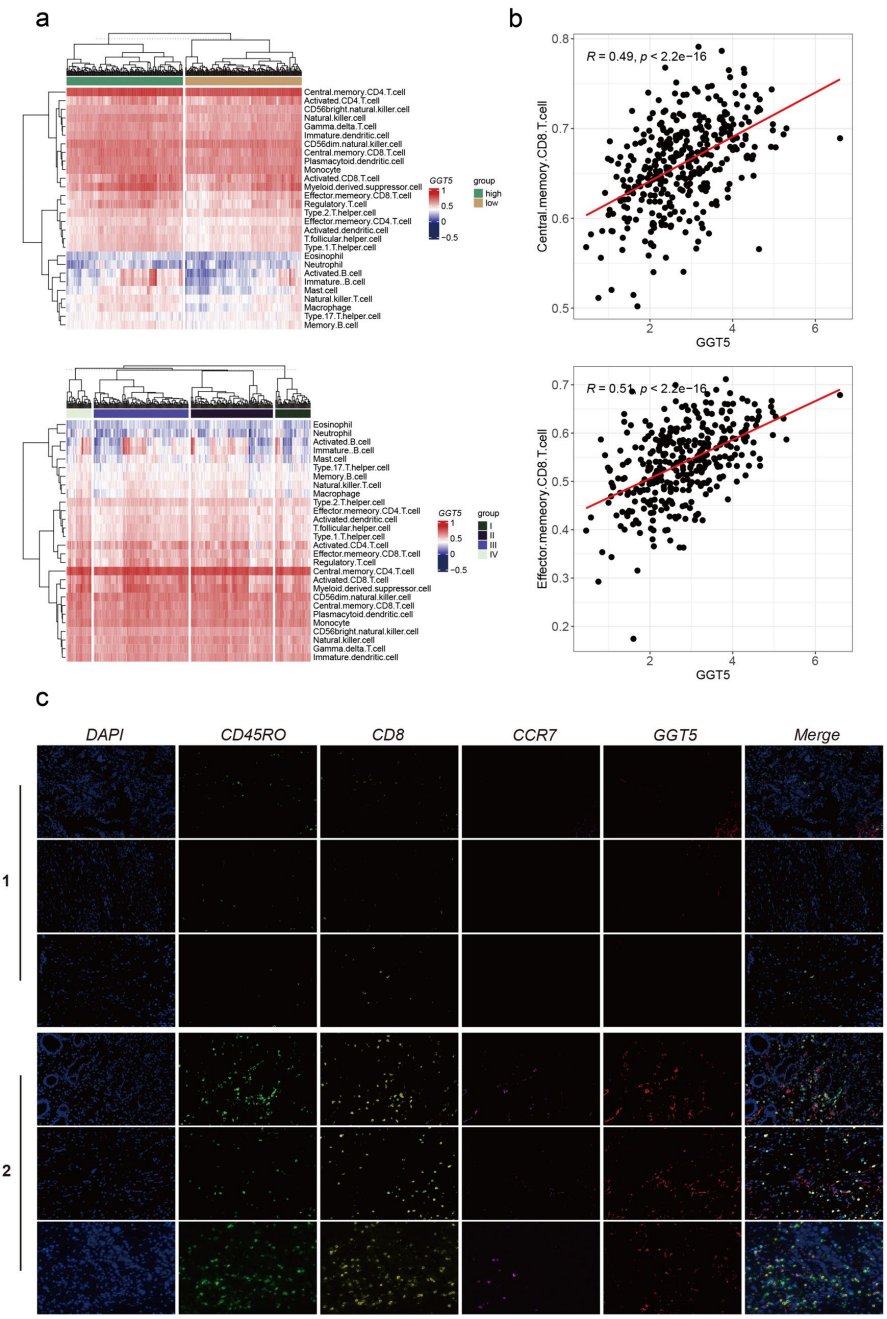
**a** ssGSEA analysis of 28 kinds of immune cells in high/low expression of *GGT5* and different stage of GC patients, suggesting that memory *CD8*+ T cells, especially TCM and TEM, are more prevalent in *GGT5* high tumors and advanced stages of GC, **b** the correlation of *GGT5* with TCM and TEM, showing a positive association with TCM and TEM, **c** mIHC analysis shows expression level of *CD45RO*, *CD8*, *CCR7*, *GGT5* in GC patients receiving immunotherapy. Group 1 represents 3 patients which are evaluated as PR according to RECIST. Group 2 represents patients which are evaluated as non-PR. mIHC expression analysis for *CD45RO*, *CD8*, *CCR7*, and *GGT5* reveals that the tumors of GC patients non-responsive to immunotherapy have enhanced expression of *GGT5* and memory *CD8*+ T cell markers. Magnification, × 400

To further interrogate the association between *GGT5* and *CD8*+ T cells, we obtained markers for 28 immune cell types and conducted ssGSEA analysis to explore the interplay among these immune cells, *GGT5*, and gastric cancer tumor stage using the TISIDB database (Fig. 8a). Our analysis revealed that higher *GGT5* expression and advanced tumor stage were linked to increased enrichment of memory *CD8*+ T cells. Furthermore, correlation analysis revealed a robust positive relationship between *GGT5* expression and central memory *CD8*+ T (TCM) cells, along with effector memory *CD8*+ T (TEM) cells (Fig. 8b).

### Multiplex immunohistochemistry (mIHC) analysis of GC tissue specimens

Multiplex immunohistochemistry (mIHC) analysis was performed among 10 GC tissue specimens to further explore the association between *GGT5* expression with memory *CD8*+ T cells and immunotherapy. The response of all the 10 patients to immunotherapy was documented (Table S2). The most representative mIHC staining images of *GGT5*, *CD8*, *CD45RO*, and *CCR7* are shown in Fig. 8. Multiplexed fluorophore-stained slides of 4 other patients were not presented due to the markedly low expression of each maker and diminished fluorescence intensity. Patients in Group 1 (patient1, 9, 10) were categorized as PR according to the RECIST, and patients in Group 2 (patient 2, 3,7) were designated as non-PR. Obviously, *GGT5* expression displayed a positive correlation with memory *CD8*+ T cells. Furthermore, patients with typically poorer responses to immunotherapy (Group 2) exhibited higher levels of *GGT5* expression and a greater concentration of memory *CD8*+ T cells. Hence, our mIHC results not only confirmed the significant correlation between *GGT5* and memory *CD8*+ T cell enrichment but also revealed that patients with elevated *GGT5* expression had a diminished response to immunotherapy.

## Discussion

Reactive oxygen species (ROS) exert severe damage on DNA in the cell [25, 26]. As an adaptive response, tumor cells possess high levels of molecules that scavenge ROS. *GSH* metabolism plays an antioxidant role that helps to eliminate free radicals and detoxify agents, and is commonly elevated during oxidative stress [27]. Disturbances in *GSH* metabolic homeostasis play a crucial role in the occurrence and progression of diseases, including cancers [28]. Furthermore, *GSH* metabolism can promote metastasis and is positively associated with therapeutic resistance in various cancer types [27, 29, 30]. Therefore, *GSH* metabolism has become a crucial target for cancer research and treatment.

In this study, genes related to *GSH* metabolism were screened to identify the active metabolic gene. *GGT5* was finally identified as the hub gene of *GSH* metabolism and predicted to regulate the TME of GC. The mechanism of how *GGT5* affects GC was explored at both the bulk RNA and scRNA levels. We found that: (1) *GGT5* is an independent prognostic gene marker for GC and can promote the tumor microenvironment; (2) *GGT5* has a highly positive connection with immune-related genes, and high expression of *GGT5* can alleviate the effect of ICIs on cancers; (3) *GGT5* plays its role mainly by promoting memory CD8+ T cells.

The *GGT* family acts in a crucial role in extracellular metabolism of *GSH*, which can maintain the balance of *GSH* metabolism via cleaving *GSH* peptides. It has been suggested that *GGT* family is elevated in multiple cancers and can lead to poor clinic outcome, such as stomach, ovarian, pancreatic, breast cancer [31–34]. Moreover, an accumulation of evidence suggests that *GGT* family plays an important role in tumorigenesis and progression of stomach cancer [35]. Not only is *GGT5* associated with poor outcome in GC, but also with the ability to regulate the TME [36, 37]. In this study, *GGT5* expression was measured based on TCGA and found to have higher expression in GC compared to normal tissues. Moreover, higher *GGT5* expression in gastric cancer is correlated with poor clinical outcomes based on OS, DFI, PFI, and DSS analyses. Furthermore, the clinical correlation analysis revealed that *GGT5* expression is elevated in higher grades of gastric cancer, such as histologic grade 3 and T stages III-IV. These findings suggest an indispensable role of *GGT5* in tumor invasion. However, the specific mechanisms of how *GGT5* affects GC is still unclear.

It has been largely noted that in response to pathogens or dangerous environments, immune cells release leukotrienes (LTs) to defend against damage [38]. *GGT5*, a γ-glutamyl leukotrienes, can metabolize *LTC4* to *LTD4* [39, 40], making it a key player in the active role of asthma pathogenesis [41] and indicating that it serves a significant role in the immune response. TIDE analysis predicted that high *GGT5* expression is associated with a poorer response to ICIs in gastric cancer, prompting further investigation into how *GGT5* affects the TME of GC. In this study, the relationship between *GGT5* and immune genes of GC was investigated, revealing a positive relation between *GGT5* and immune-active genes and chemokine receptors, particularly *CXCL12*, *CXCR4*, and *TGFB1*. *CXCL12* is a small protein that binds with *CXCL4* on tumor cells, triggering the anti-apoptosis pathway via Bcl-2 upregulation signal pathways and the EMT pathway via the Rho-ROCK pathway [42]. Elevated expression of *CXCR4* and/or *CXCL12* expression has been examined in larger tumors and lymphatic invasion samples in gastric cancer [43], as well as decreased response to radio- and chemo-therapy in various cancer types [44]. *TGFB1* facilitates the progression and evasion of cancers, suppressing antitumor immune responses. *TGFB1* has been shown to play a role in the immune escape of gastric cancer, with the mechanism likely involving the suppression of *CD8*+ T cells and increases in regulatory T (Treg) cells [45, 46]. Therefore, *GGT5* may exert a pro-tumor effect by regulating immune genes such as *CXCL12* and *CXCL4* in the tumor microenvironment. This finding prompts further investigation into the involvement of immune cells. In tumor metabolism, *GGT5* exhibits catalytic activity in both hydrolysis and transpeptidation reactions. As one of the few extracellular enzymes identified with the capability to cleave gamma-glutamyl bonds, *GGT5* assumes a significant role in the initial stage of glutathione metabolism, involving the cleavage of gamma-glutamyl bonds [47]. Consequently, attenuating the expression of *GGT5* would impede GSH metabolism and enhance anti-tumor capabilities. The application of GGsTOP, a remarkably selective and potent irreversible inhibitor of GGT activity, can markedly lower the expression of *GGT5*, hinder the proliferation and chemotherapy resistance of lung adenocarcinoma cells [48]. These findings underscore the importance of *GGT5* as a novel target for anti-tumor therapy. Exploration of the *GGT5* amino acid sequence reveals distinctive residues that interact with the glutathione active site, providing a theoretical groundwork for the future development of drugs targeting *GGT5* [49].

In our study, CIBERSORT analysis revealed the infiltration of 8 kinds of immune cells between different expression group of *GGT5* in gastric cancer. To better understand the underlying mechanism of *GGT5* and TME, further investigation of the relationship between *GGT5* and these immune-related cells is necessary. The correlation analysis indicated that *GGT5* has a positive correlation with *CD8* T cell, Tregs, B cells naïve, monocytes, and resting Mast cells, while it has a negative correlation with resting *CD4* memory T cells. Similar trend was found in EPIC, MCP-counter, xCell, and ESTIMATE analysis. It has been reported that *CD8*+ T cells play a crucial role in tumor immune resistance with *PD-L1*, and increases in *CD8*+ T cells are associated with poor PFS and OS of gastric cancer [50]. Tregs, promoted by the *TNFR2* signaling pathway, are identified as immune suppressors, and their accumulation in the TME of GC is considered a risk factor in prognosis [51]. Also, the infiltration of *CD4*+ T cells is higher in patients with T3 stage of GC compared to that in T1 and T2 stage, implying a critical effect of *CD4*+ T cells in the prognosis of gastric cancer [52]. We next conducted scRNA analysis to identify the heterogeneity of immune cell types within the TME of gastric cancer, which were initially clustered into eight cell types. By combining the results of immune infiltration and scRNA analysis, we explored the specialty of T cells, which prompted further investigation. A previous study demonstrated that *GSH* metabolism is dispensable for early T cell activation but can promote T cell growth [53]. In our study, we evaluated the involvement of *GSH* metabolism in T cells and subsequently identified characteristic T cell subsets. Our results indicated that *GGT5* is mainly involved in three *CD8*+ T cell subsets named *C0-CD8-IL7R*, *C5-CD8-CCR7*, and *C8-CD8-TK1*. Furthermore, we isolated *CD8*+ T cells and *CD4*+ T cells; it was found that *GGT5* was extremely higher in *CD8*+ T cells than that in *CD4*+ T cells. These results exhibit a strong correlation with *CD8*+ T cells. According to previous studies, the loss of *IL-7* and *IL-7R* can lead to severe immune deficiency, and *IL-7R* acts a vital role in the generation and long-term maintenance of memory *CD8*+ T cells [54]. Deficiency in *CCR7* can impair the function of memory *CD8*+ T cells to sustain *IL-7* and *IL-7R*, which has a negative effect on the survival and homeostasis of memory *CD8*+ T cells [55]. *TK1*, which is associated with *CD8*+ T cells [56], is a marker of abnormal tumor cell proliferation. Its concentration increases with lesion growth, disease progression, and distant metastasis [57]. In summary, our findings indicate a connection between *GGT5* and memory *CD8*+ T cells. Next, we investigated *GGT5* and 28 immune cell types based on ssGSEA analysis. Our findings demonstrated that memory *CD8*+ T cells, including TCM and TEM, were more abundant in the group with higher *GGT5* expression and in the higher tumor stage group. This suggests a role for *GGT5* in maintaining *CD8*+ T cells, especially TCM and TEM cells.

Three kinds of memory T cells have now been reported in peripheral blood: central memory T cells (TCM) located in secondary lymphoid tissues and reactivated during secondary infection; effector memory T cells (TEM) that circulate in various tissues and are cytotoxic; and tissue-resident memory T cells (TRM) that persist in tissues without circulating [58, 59]. It has been shown that cancer patients treated with ICIs show an increasing frequency of memory T cells, both TCM and TEM cells, and that infiltration of memory *CD8*+ T cells is associated with poor prognosis [60, 61]. Our findings in this study are consistent with these previous results. We conducted mIHC analysis to investigate the expression mode of *GGT5* in GC tissues and its correlation with the response to immunotherapy and found that patients with a poor response to immunotherapy have higher *GGT5* expression and more memory *CD8*+ T cell accumulation. In addition, our analysis using monocle revealed that memory T cell clusters, specifically *C0-CD8-IL7R* and *C5-CD8-CCR7*, predominantly accumulate in the early and middle stages of T cell differentiation. Unlike effector T cells, memory T cells do not fully transition but persist, contributing to long-term immunological memory. Consequently, memory T cells are positioned within the intermediate stage of *CD8*+ T cell differentiation, according to the linear model, owing to their shared characters of transcriptional, phenotypic, and epigenetics with both effector and naïve T cells [62]. Notably, the impact of *GGT5* on T cells has not been previously reported, and our findings indicate that the expression of *GGT5* mostly accumulates during the early and middle period of the T cell trajectory, mirroring the distribution of memory *CD8*+ T cells. This finding implies a potential role for *GGT5* in sustaining the longevity of memory *CD8*+ T cells throughout the progress of gastric cancer.

Previous studies have already shown that a reduction in *GSH* levels and a rise in ROS levels can impair the formation and maintenance stage of memory *CD8* T cells. In contrast, exogenous GSH can shield TCM and TEM cells from apoptosis [63, 64]. Nevertheless, the connection between *GGT5*, an active gene in GSH metabolism, and memory *CD8*+ T cells has rarely been explored and reported. Our study is the first to propose that *GGT5* wields a remarkable effect on the initiation and progression of gastric cancer, while concurrently regulating the immune milieu by preserving live memory *CD8*+ T cells. Nevertheless, our study still has several shortcomings, which are detailed below: (1) Future clinical investigations involving multiple centers and extensive cohorts are imperative to validate this predictive signature before deeming it clinically beneficial; (2) additional experiments, including both overexpression and knockdown studies, as well as immune/tumor co-culture experiments, are essential to clarify the functional impact of *GGT5* on the development and progression of gastric cancer; (3) future progress of foundational experiments concerning molecular structure research and the exploration of *GGT5*'s druggable potential is crucial for its potential application in clinical therapy.

## Conclusion

This study shows that *GGT5* serves as an active gene of GSH metabolism and promotes progression, drug resistance, and immune microenvironmental remodeling in gastric cancer. Although some of these processes have been reported before, the specific mechanism of how this occurs still remain unknown. Moreover, the interplay between *GGT5* and memory *CD8*+ T cells and their influence on gastric cancer has rarely been studied. A deeper comprehension of the role of *GGT5* in these pathways may offer valuable insights for the treatment of gastric cancer.

### Supplementary Information

Below is the link to the electronic supplementary material.

Supplementary file1 (ZIP 1073 kb)

## Data Availability

All experimental data required to prove the conclusions of the study have been included in this paper and/or the supplemental materials. Other data relating to our study could be accessible from the corresponding author. Publicly supporting data from TCGA could be downloaded from https://xenabrowser.net/. GEO data are available from GSE167297 and GSE15459.

## Abbreviations

GC: Gastric cancer
GSH: Glutathione
ICIs: Immune checkpoint inhibitors
ROS: Reactive oxygen species
TCM: Central memory CD8+ T cells
TEM: Effector memory CD8+ T cells
TME: Tumor microenvironment
