# Supplementary material for: GGT5: a potential immunotherapy response inhibitor in gastric cancer by modulating GSH metabolism and sustaining memory CD8+ T cell infiltration
Source: Cancer Immunol Immunother. 2024 May 15;73(7):131. doi: 10.1007/s00262-024-03716-3 (PMC11096297; doi:10.1007/s00262-024-03716-3)
Supplement: Supplementary file 1 — Supplementary file1 (ZIP 1073 kb) [file 262_2024_3716_MOESM1_ESM.zip › Supplementary Matierals/Supplementary Matierals-Figures.pdf]

GGT5: A Potential Immunotherapy Response Enhancer in Gastric Cancer by Modulating GSH Metabolism and Sustaining Memory CD8+ T Cell Infiltration

Wenjing Zhao[1+], Ziwei Liang[1+], Yongshi Yao[1], Yang Ge[1], Guangyu An[1], Ling Duan[2], Jiannan Yao[1*]

Affiliation:

[1]Beijing Chaoyang Hospital, Capital Medical University, Beijing, China

2Beijing Tiantan Hospital, Capital Medical University, Beijing, China

***Correspondence:**

Jiannan Yao, yaojiannan@mail.ccmu.edu.cn

ORCID: https://orcid.org/0000-0002-4982-4460

Supplementary Materials

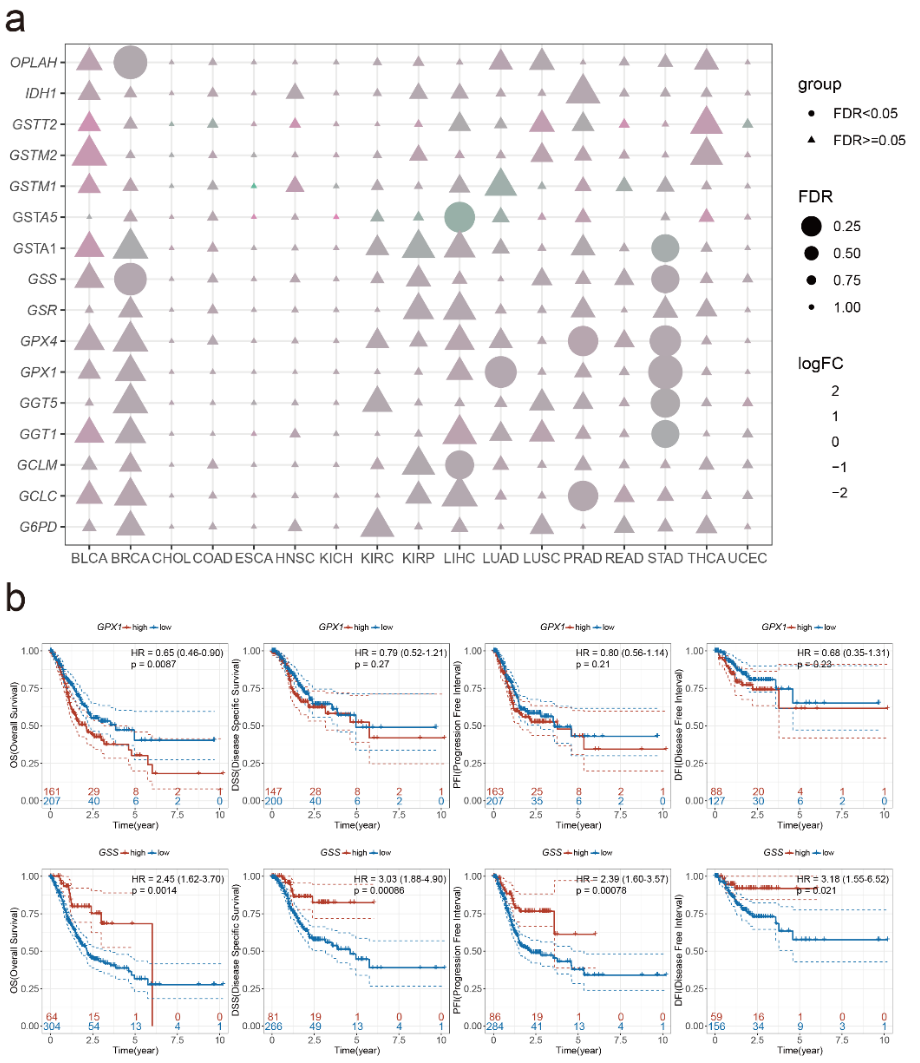

Fig.S1 Expression pattern and survival analysis of glutathione metabolic genes **a)** The bubble plot shows the expression pattern of glutathione metabolism in tumor and normal tissues across 17 kinds of cancer types. The cancer types with less than 5 normal tissues paired were excluded in this study. The point size represents FDR. The color represents fold-change. The shape represents if the FDR value <0.05 **b)** The gastric cancer samples were divided into high and low groups of *GSS* and *GPX1* expression according to the maxstat method. The Kaplan-Meier curves of survival analysis including overall survival analysis (OS), progression free interval analysis (PFI), disease free interval analysis (DFI) and disease specific

survival analysis (DSS) were plotted. It shows that high expression of *GSS* has a better OS, DFI, PFI and DSS, with no meaning of PFI and DFI. Moreover, it shows that high expression of *GPX1* has a better DFI (p=0.0087), with no meaning of OS, PFI, DSS

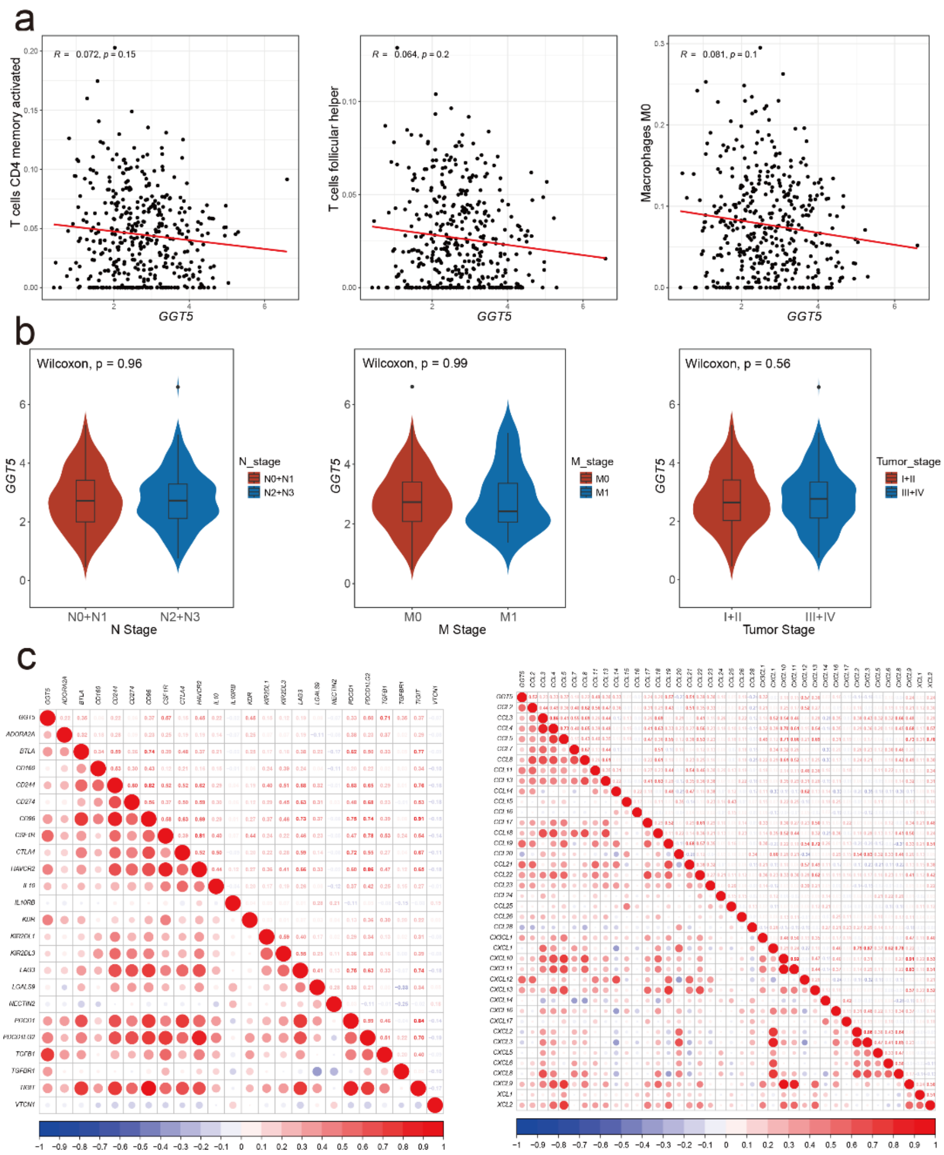

Fig.S2 Correlation analysis of *GGT5* with immune cells and clinicopathological characters **a)** The correlation analyses show that *GGT5* has no statistical meaning with T cells *CD4* memory activated, T cells follicular helper and Macrophages M0 **b)** The correlation between *GGT5* and clinicopathological characters of gastric cancer show that there is no significant statistical meaning between *GGT5* and tumor stage, N stage and M stage **c)** The bubble plots represent the relationship of *GGT5* with immune-suppressive genes and chemokines. It shows that *GGT5* is positively associated with most of immune-related genes. The color and size of the dots represent the correlation coefficient

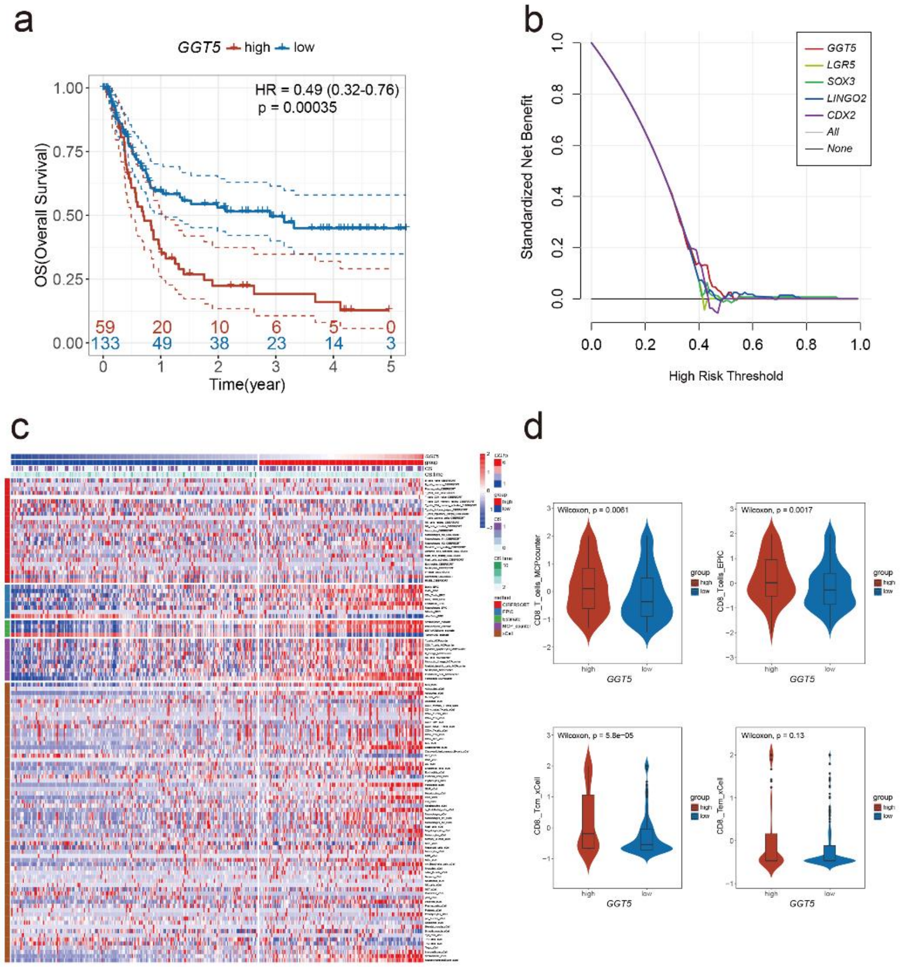

Fig.S3 The prognosis model and immune cell infiltration of *GGT5* **a)** Gastric cancer patients RNA-seq data and corresponding survival data was collected from GSE15459. Patients are divided into high/low groups according to the optimal cut-off value of *GGT5*. The result shows that higher expression of *GGT5* has a poor clinic outcome (p=0.00035, HR = 0.49 (0.32-0.76)) **b)** DCA curves of *GGT5* and another four predictable model of clinic benefit of gastric cancer. It displays that *GGT5* has better clinic outfit **c-d)** The heatmap displays differential immune infiltration mode of *GGT5* in gastric cancer. The violin plot presents that *CD8*+T cells were observed to have significantly higher expression levels in the *GGT5* high expression group
